# Construction and Analysis of the Tumor-Specific mRNA–miRNA–lncRNA Network in Gastric Cancer

**DOI:** 10.3389/fphar.2020.01112

**Published:** 2020-07-21

**Authors:** Xiaohao Zheng, Xiaohui Wang, Li Zheng, Hao Zhao, Wenbin Li, Bingzhi Wang, Liyan Xue, Yantao Tian, Yibin Xie

**Affiliations:** ^1^Department of Pancreatic and Gastric Surgery, National Cancer Center/National Clinical Research Center for Cancer/Cancer Hospital, Chinese Academy of Medical Sciences and Peking Union Medical College, Beijing, China; ^2^Department of General Surgery, Xuanwu Hospital, Capital Medical University, Beijing, China; ^3^Department of General Surgery, The First People’s Hospital of Dongcheng District, Beijing, China; ^4^Department of Cardiovascular Surgery, China-Japan Friendship Hospital, Beijing, China; ^5^Department of Pathology, National Cancer Center/National Clinical Research Center for Cancer/Cancer Hospital, Chinese Academy of Medical Sciences and Peking Union Medical College, Beijing, China

**Keywords:** gastric cancer, weighted correlation network analysis, competing endogenous RNA, risk score, The Cancer Genome Atlas, Genotype-Tissue Expression, Gene Expression Omnibus

## Abstract

Weighted correlation network analysis (WGCNA) is a statistical method that has been widely used in recent years to explore gene co-expression modules. Competing endogenous RNA (ceRNA) is commonly involved in the cancer gene expression regulation mechanism. Some ceRNA networks are recognized in gastric cancer; however, the prognosis-associated ceRNA network has not been fully identified using WGCNA. We performed WGCNA using datasets from The Cancer Genome Atlas (TCGA) and the Genotype-Tissue Expression (GTEx) to identify cancer-associated modules. The criteria of differentially expressed RNAs between normal stomach samples and gastric cancer samples were set at the false discovery rate (FDR) < 0.01 and |fold change (FC)| > 1.3. The ceRNA relationships obtained from the RNAinter database were examined by both the Pearson correlation test and hypergeometric test to confirm the mRNA–lncRNA regulation. Overlapped genes were recognized at the intersections of genes predicted by ceRNA relationships, differentially expressed genes, and genes in cancer-specific modules. These were then used for univariate and multivariate Cox analyses to construct a risk score model. The ceRNA network was constructed based on the genes in this model. WGCNA-uncovered genes in the green and turquoise modules are those most associated with gastric cancer. Eighty differentially expressed genes were observed to have potential prognostic value, which led to the identification of 12 prognosis-related mRNAs (*KIF15, FEN1, ZFP69B, SP6, SPARC, TTF2, MSI2, KYNU, ACLY, KIF21B, SLC12A7*, and *ZNF823*) to construct a risk score model. The risk genes were validated using the GSE62254 and GSE84433 datasets, with 0.82 as the universal cutoff value. 12 genes, 12 lncRNAs, and 35 miRNAs were used to build a ceRNA network with 86 dysregulated lncRNA–mRNA ceRNA pairs. Finally, we developed a 12-gene signature from both prognosis-related and tumor-specific genes, and then constructed a ceRNA network in gastric cancer. Our findings may provide novel insights into the treatment of gastric cancer.

## Introduction

Gastric cancer is a major cause of cancer-related mortality worldwide (Van Cutsem et al., 2016). It is a serious form of cancer characterized by limited chemotherapy regimens and complex patterns of tumorigenesis and progression in different subtypes (Erdem et al., 2018; Kubota et al., 2020). There have been exceptional advancements in the interpretation of the molecular pattern of gastric cancer through research projects including the Cancer Genome Atlas (TCGA) (Cancer Genome Atlas Research N, 2014) and the Asian Cancer Research Group (ACRG) (Cristescu et al., 2015) in recent years; however, current classifications are not sufficient to describe the vast differences in prognoses and summarize overall genomic characteristics, even for patients who are recognized as belonging to the same molecular subtypes.

Integrated analysis of transcriptomes is believed to provide peculiar insights into diseases; in this respect, weighted gene co-expression network analysis (WGCNA) may be the most popular approach for detecting co-expressed RNAs from RNA-seq data to microarray data (van Dam et al., 2018; Kakati et al., 2019). WGCNA can identify select groups of significant genes with similar biological functions and with strong correlations to specific traits. Many recent surveys have used WGCNA for both non-neoplastic and neoplastic diseases, including gastric cancer.

Salmena et al. (2011) speculated that the expression of many RNA transcripts is regulated by competing endogenous RNAs (ceRNAs) competing for the same sequences in miRNAs. They established the groundwork for a significant discovery of a communication network between coding and non-coding RNAs. Their theory has been supported by studies on the pathological processes of many malignancies, including breast, colon, and gastric cancers (Qi et al., 2015; Shuwen et al., 2018; Abdollahzadeh et al., 2019).

Thus, finding key genes that will serve as drug targets is crucial to the treatment of gastric cancer. In this study, we used WGCNA to construct a solid cancer-associated ceRNA network in gastric cancer for the first time. We hypothesized that identifying gene co-expression patterns would provide additional insight into disease-associated biological pathways. Finally, we explored a lncRNA–miRNA–mRNA network based on the survival-related hallmark genes of gastric cancer, providing candidate targets for its management and surveillance.

## Materials and Methods

### RNA-Sequencing and Microarray Data Collection

RNA-seq data were obtained from TCGA repository (https://portal.gdc.cancer.gov/) and the Genotype-Tissue Expression (GTEx) portal (https://www.gtexportal.org/) and sequenced on the Illumina HiSeq 2000 RNA Sequencing platform. TCGA offers a comprehensive database of cancer genomic profiles of specific cancer types. GTEx is another project that recruits postmortem donors without diseases, which has made genetic traits of healthy people open to the public. We performed a combined analysis of the stomach data from these two projects in the present study. The transcript per million (TPM) expression values for 625 stomach samples and the RNA-seq by Expectation Maximization (RSEM) expected counts for 624 stomach samples were preserved for further analysis (raw counts data of a patient did not exist).

The microarray data were downloaded from the Gene Expression Omnibus (GEO, http://www.ncbi.nlm.nih.gov/geo/). The datasets were obtained from human gene expression microarray profiles of gastric cancer using fresh-frozen specimens. Finally, we included GSE62254 and GSE84433 for further analysis, which are the two largest datasets of microarray-based gene expression profiling. GSE62254 was profiled on the Affymetrix Human Genome U133 Plus 2.0 Array platform (Affymetrix, Inc., Santa Clara, CA, USA), including 300 gastric cancer tumor samples and 100 normal samples, which is the largest number of normal gastric samples among datasets (Cristescu et al., 2015).

We downloaded raw data (CEL files) generated by the Affymetrix platform. The R package oligo was utilized for format conversion, missing data filling, background correction, and data normalization (Parrish and Spencer , 2004). GSE84433 was profiled on the Illumina HumanHT-12 V3.0 Expression Beadchip (Illumina, Inc., San Diego, CA, USA); it includes 357 gastric cancer samples, and thus has the highest number of gastric cancer patients with survival information (Cheong et al., 2018). We downloaded data in the format of raw counts, performed quantile normalization, followed by log transformation.

Annotation of the above datasets was performed according to the different platforms using the official R package downloaded from Bioconductor (http://www.bioconductor.org/packages/). When the same RNA name appeared, the probe with the highest signal value was stored. To facilitate the analysis, only the overlapped RNAs qualified for survival-related ceRNA network construction. To keep the data updated, clinical and survival information of patients were obtained from the websites on March 5th, 2020. All original data were retrieved from the open database; thus, the documents of medical ethics were exempted as all had been approved when first published.

### Differentially Expressed RNAs

The Limma package was used for screening differentially expressed RNAs (Ritchie et al., 2015). The RSEM expected counts retrieved from the GTEx database and TCGA database were utilized to discern differentially expressed RNAs between gastric cancer and control groups, which comprised normal stomach tissue both from dead non-cancerous subjects and adjacent stomach tissue in gastric cancer patients. For microarray-based profiling, expression values retrieved from GSE62254 were utilized to distinguish differentially expressed RNAs between 300 gastric cancer samples and 100 normal tissue samples. The R package Limma was used to process the data with the standard of false discovery rate (FDR) < 0.01, and |fold change (FC)| > 1.3. After filtering out RNAs with low expression values, the overlapping, differentially expressed RNAs were considered suitable for further analysis.

### WGCNA

WGCNA is a bioinformatics method for dealing with high-throughput gene expression data, which can be used for the construction of a co-expression network (Langfelder and Horvath, 2008). The expression values of genes were preprocessed in the form of log2 (TPM + 0.001). The genes were then chosen in order of descending variance of their expression in the datasets. Finally, 13000 genes were put through WGCNA. Pairwise Pearson coefficients were used to assess the weighted co-expression relationships between all genes to produce an adjacency matrix. The least value for which the scale-free topology fit R^2 index > 0.75 was chosen as the soft-threshold power. Pearson coefficients were produced for all paired genes; thus, the co-expression matrix was rendered into an adjacency matrix using soft-threshold power. The soft-threshold power was selected according to the standard scale-free distribution. Scale-free co-expression networks were created with 30 RNAs as the minimal module size and 0.25 as the dendrogram cut height for module merging. The soft threshold was used to ensure a scale-free network. Genes with high correlations were clustered into the same module after forming a co-expression network.

### Gene Function Analysis

Upregulated and downregulated differentially expressed genes were put into Gene Ontology (GO) and Kyoto Encyclopedia of Genes and Genomes (KEGG), respectively. For the gene set enrichment analysis (GSEA) and KEGG–GSEA, all genes were incorporated into the analysis. A pathway (term) with an adjusted p-value < 0.05 was considered a functional enriched pathway (term) using the R package ClusterProfiler (Yu et al., 2012).

### ceRNA Network

RNAinter was employed to observe the relationships among mRNA, lncRNA, and miRNA. RNAinter contains 24 databases demonstrated by experiment and 14 databases forecasted by calculation, including miRTarBase and starBase. We used lncRNA–miRNA relationships and mRNA–miRNA relationships with the least confidence score limit of 0.5 for further analysis (Lin et al., 2020). To ensure mRNA–lncRNA competing relationship pairs that have the shared miRNAs in gastric cancer, the Pearson correlation test and the hypergeometric test were utilized using the expression profiles. Quantile normalization and correction of batch effects between TCGA dataset and the GTEx dataset were performed for the expression profiles before further analysis (Leek et al., 2012). If the *P*-values of both tests were less than 0.05, the mRNA–lncRNA competing interaction pairs were saved to create the ceRNA network (Paci et al., 2014). The co-expression network with the overlapped lncRNA–miRNA–mRNA relationship was visualized with Cytoscape software (Su et al., 2014).

### Construction and Validation of the Survival Model

Training datasets for the prognostic score system were performed according to the sequencing data from TCGA. Validation of the prognostic score system was performed on the two microarray datasets (GSE62254 and GSE84433). Patients who died within 30 postoperative days and without 30-day follow-up were excluded. Expression profiles of eligible gastric cancer patients were normalized and non-overlapping genes were removed from the analysis. Before survival analysis, the gene expression values in the datasets were processed with a standardization with 0 mean value and standard deviation of 1. Based on the mRNAs obtained in the clinical cancer-associated modules, univariate Cox regression analysis was performed to identify prognosis-related mRNAs; then, all the survival-related genes were used to perform multivariate Cox regression. The backward selection method was used to select the most suitable survival gene group to construct a risk score system (Donithan et al., 1992). The samples in the datasets were divided into high-risk and low-risk groups according to the universal cutoff value of their risk scores. Kaplan–Meier survival curves were used to evaluate correlations between the overall survival of the two groups in datasets using the survival package (Therneau, 2020).

## Results

### Differential Gene Expression Analysis

**Figure 1:**
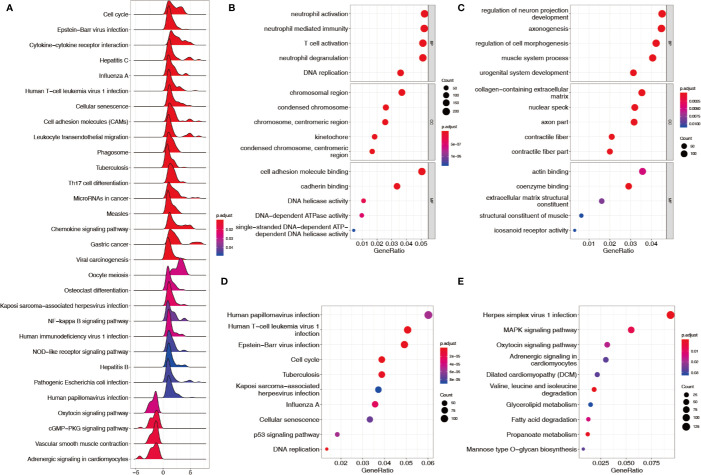
Enrichment analysis of KEGG–GSEA, GO, and KEGG. **(A)** KEGG–GSEA after filtering out low-expressed genes. **(B)** GO terms enriched from upregulated genes. **(C)** GO terms enriched from downregulated genes. **(D)** KEGG pathways enriched from upregulated genes. **(E)** KEGG pathways enriched from downregulated genes.

Sequencing data (TCGA + GTEx) and microarray data (GSE62254) were downloaded and processed as previously described. A total of 13007 genes and 292 lncRNAs were identified after screening out less-expressed ones. Thirty pathways were generated after the genes were applied to the KEGG–GSEA (**Figure 1A**). A total of 3895 differentially expressed genes and 79 lncRNAs were determined by comparing each gastric cancer group to both control groups. In total, 2347 upregulated and 1548 downregulated genes were subjected to GO term and KEGG pathway enrichment analyses. In the GO analysis for upregulated genes, neutrophil activation, neutrophil mediated immunity, and T cell activation were rendered as the top three terms of biological processes (BP); the chromosomal region, condensed chromosome, and centromeric region on chromosome were rendered as the top three terms of cellular components (CC); cell adhesion molecule binding, cadherin binding, and DNA helicase activity were rendered as the top three terms of molecular functions (MF) (**Figure 1B**). In the GO analysis for downregulated genes, regulation of neuron projection development, axonogenesis, and regulation of cell morphogenesis were rendered as the top three terms of the BP; the collagen-containing extracellular matrix, nuclear speck, and axon part were rendered as the top three terms of CC; actin binding, coenzyme binding, and extracellular matrix structural constituent were rendered as the top three terms of the MF (**Figure 1C**). In the KEGG analysis, human papillomavirus infection, human T−cell leukemia virus 1 infection, and Epstein−Barr virus infection were rendered as the top three enriched pathways for upregulated genes (**Figure 1D**); herpes simplex virus 1 infection, MAPK signaling, and oxytocin signaling were rendered as the top three pathways enriched for downregulated genes (**Figure 1E**).

### WGCNA

**Figure 2:**
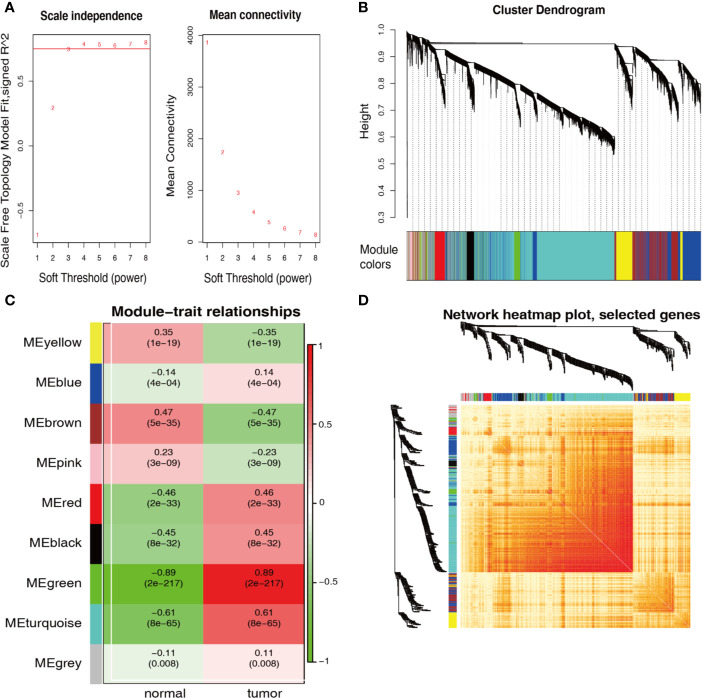
WGCNA identification of cancer-associated RNA modules. **(A)** Graphs of soft-threshold power versus scale-free topology model Fit index and mean connectivity. Four was chosen as the appropriate soft-power. **(B)** Cluster dendrogram of the co-expression network modules created according to the dissimilarity of the topological overlap in the selected mRNAs. **(C)** Analysis of relationships between genes in modules between gastric cancer and normal samples. The green and turquoise modules were the most tumor-specific modules. **(D)** Heatmap plot of topological overlap in the mRNA network. Selected genes in the green and turquoise modules showed higher topological overlap. The gene dendrogram and the corresponding module are shown along the left and top.

WGCNA was performed to ascertain the most strongly cancer-associated genes. When the soft-power β was set to 4, the scale-free topology fit index was over 0.75 (**Figure 2A**). The created network included nine modules (**Figure 2B**). **Figure 2C** shows that the green module was recognized as the most specific module with a coefficient of correlation of 0.89 (p = 2 × 10^−217^); the turquoise module came in second with a coefficient of correlation of 0.61 (p = 8 × 10^−65^). Genes in both modules showed a high correlation with each other according to the heatmap of the topological overlap plot (**Figure 2D**).

### Functional Analysis of the Green and Turquoise Modules

**Figure 3:**
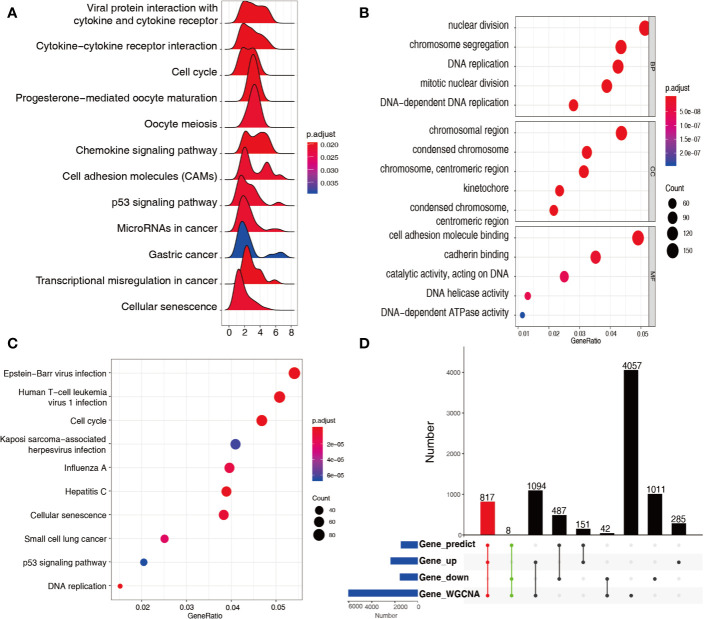
Functional analysis of genes in green and turquoise modules. **(A)** KEGG–GSEA for genes in the green and turquoise modules for signaling pathway analysis. **(B)** GO terms enriched from upregulated genes in the green and turquoise modules. **(C)** KEGG pathways enriched from upregulated genes in the green and turquoise modules. **(D)** 825 mRNAs recognized at the intersection of genes predicted, differentially expressed genes, and genes in the green and turquoise modules.

A total of 805 genes in the green module and 5213 genes in the turquoise module were subjected to KEGG–GSEA, generating 12 pathways (**Figure 3A**). The GO and KEGG enrichment results of 3312 upregulated differentially expressed genes in both modules are plotted in **Figures 3B, C**, in which enrichment results of the downregulated differentially expressed genes were not found. In total, 817 upregulated and eight downregulated differentially expressed genes were found to be qualified, with appropriate ceRNA relationships in the stomach, and appeared in the two cancer-associated modules (**Figure 3D**).

### Survival Model Construction and Validation

The clinical and pathological data from the construction and validation cohorts are shown in **Table 1**. We used Cox univariate regression analysis for the 825 genes for identifying survival-related genes in 332 TCGA gastric cancer samples. The univariate analysis screened 83 predictors based on prognosis. Because three genes did not appear in GSE84433, 80 genes were included in the multivariate analysis, which further led to the identification of 12 upregulated mRNAs for constructing a risk score model, containing *KIF15, FEN1, ZFP69B, SP6, SPARC, TTF2, MSI2, KYNU, ACLY, KIF21B, SLC12A7*, and *ZNF823* (**Table 2**). The risk scores for individual samples was calculated using the formula:

**Table 1:** The baseline characteristics of the construction and validation cohorts.

Variables	TCGA			GSE622554			GSE84433		
	Alive	Dead	N (%)	Alive	Dead	N (%)	Alive	Dead	N (%)
**Age**									
>=60	122 (64.6)	104 (74.3)	226 (68.7)	91 (61.5)	103 (67.8)	194 (64.7)	85 (46.4)	114 (66.3)	199 (56.1)
<60	67 (35.4)	36 (25.7)	103 (31.3)	57 (38.5)	49 (32.2)	106 (35.3)	98 (53.6)	58 (33.7)	156 (43.9)
**Gender**									
male	117 (60.9)	97 (69.3)	214 (64.5)	99 (66.9)	100 (65.8)	199 (66.3)	117 (63.9)	123 (71.5)	240 (67.6)
female	75 (39.1)	43 (30.7)	118 (35.5)	49 (33.1)	52 (34.2)	101 (33.7)	66 (36.1)	49 (28.5)	115 (32.4)
**pT**									
T4	49 (25.5)	38 (27.9)	87 (26.5)	5 (3.4)	16 (10.5)	21 (7.0)	107 (58.5)	135 (78.5)	242 (68.2)
T3	84 (43.8)	70 (51.5)	154 (47.0)	30 (20.3)	61 (40.1)	91 (30.3)	39 (21.3)	28 (16.3)	67 (18.9)
T2	47 (24.5)	27 (19.9)	74 (22.6)	113 (76.4)	75 (49.3)	188 (62.7)	28 (15.3)	7 (4.1)	35 (9.9)
T1	12 (6.3)	1 (0.7)	13 (4.0)	0 (0.0)	0 (0.0)	0 (0.0)	0 (0.0)	0 (0.0)	0 (0.0)
**pN**									
N3	28 (15.1)	40 (29.6)	68 (21.2)	10 (6.8)	41 (27.0)	51 (17.0)	10 (5.5)	21 (12.2)	31 (8.7)
N2	39 (21.0)	27 (20.0)	66 (20.6)	30 (20.3)	50 (32.9)	80 (26.7)	35 (19.1)	64 (37.2)	99 (27.9)
N1	49 (26.3)	40 (29.6)	89 (27.7)	79 (53.4)	52 (34.2)	131 (43.7)	89 (48.6)	65 (37.8)	154 (43.4)
N0	70 (37.6)	28 (20.7)	98 (30.5)	29 (19.6)	9 (5.9)	38 (12.7)	49 (26.8)	22 (12.8)	71 (20.0)

**Table 2:** Univariate and multivariate Cox analyses of survival-related genes in the training group.

Gene	univariate					multivariate				
	coef	HR	lower95	upper95	p-value	coef	HR	lower95	upper95	p-value
CKAP2	-0.20	0.82	0.69	0.97	2.11E-02					
F2R	0.22	1.24	1.05	1.47	1.16E-02					
ZNF367	-0.20	0.82	0.70	0.96	1.61E-02					
ASF1B	-0.14	0.87	0.76	1.00	4.71E-02					
UHRF1	-0.18	0.84	0.72	0.98	2.31E-02					
BRIP1	-0.18	0.83	0.71	0.97	1.98E-02					
NDC1	-0.19	0.83	0.71	0.97	2.10E-02					
KIF15	-0.15	0.86	0.74	1.00	4.96E-02	0.56	1.76	1.29	2.39	3.13E-04
DCLRE1B	-0.21	0.81	0.69	0.96	1.53E-02					
TFDP1	-0.19	0.83	0.70	0.98	2.61E-02					
DCK	-0.19	0.83	0.70	0.98	3.31E-02					
RAD54L	-0.15	0.86	0.75	1.00	4.93E-02					
CTHRC1	0.25	1.28	1.08	1.52	4.82E-03					
FEN1	-0.20	0.82	0.70	0.96	1.30E-02	-0.24	0.78	0.62	1.00	4.77E-02
BCL11B	-0.21	0.81	0.69	0.96	1.28E-02					
LMNB2	-0.24	0.79	0.67	0.92	3.53E-03					
TBC1D31	-0.18	0.83	0.70	0.98	2.96E-02					
BORA	-0.18	0.83	0.71	0.97	2.06E-02					
COL10A1	0.19	1.21	1.01	1.44	3.66E-02					
GMCL1	-0.22	0.80	0.67	0.95	1.12E-02					
FGD6	0.19	1.21	1.03	1.43	2.29E-02					
ZFP69B	-0.18	0.83	0.71	0.98	2.58E-02	-0.26	0.77	0.62	0.95	1.62E-02
VCAN	0.28	1.32	1.11	1.57	1.97E-03					
E2F2	-0.19	0.83	0.72	0.96	1.22E-02					
SSX2IP	-0.22	0.80	0.67	0.96	1.44E-02					
RBBP8	-0.19	0.83	0.70	0.98	2.41E-02					
MASTL	-0.19	0.83	0.70	0.97	2.27E-02					
THY1	0.20	1.22	1.03	1.44	2.18E-02					
TIMM8A	-0.19	0.83	0.70	0.98	2.72E-02					
TMEM201	-0.17	0.84	0.71	1.00	4.86E-02					
RMI1	-0.23	0.80	0.68	0.94	6.33E-03					
LRFN4	-0.16	0.85	0.73	1.00	4.98E-02					
SLC7A1	-0.22	0.80	0.67	0.95	1.11E-02					
SLC52A3	-0.25	0.78	0.67	0.90	9.97E-04					
COA7	-0.27	0.76	0.64	0.91	1.96E-03					
COL3A1	0.19	1.21	1.02	1.43	2.83E-02					
SKIL	0.18	1.19	1.00	1.41	4.47E-02					
STRIP2	-0.26	0.77	0.65	0.92	3.64E-03					
TCF3	-0.19	0.82	0.70	0.97	2.00E-02					
PAQR4	-0.16	0.85	0.73	0.99	4.20E-02					
SP6	-0.24	0.79	0.67	0.93	4.16E-03	-0.23	0.79	0.65	0.96	1.92E-02
DPP3	-0.16	0.86	0.74	0.99	4.16E-02					
SPARC	0.26	1.30	1.09	1.54	3.21E-03	0.18	1.20	1.00	1.44	4.69E-02
ANOS1	0.18	1.20	1.01	1.43	4.24E-02					
TTF2	-0.28	0.76	0.64	0.90	1.21E-03	-0.38	0.69	0.53	0.89	3.67E-03
CCDC18	-0.22	0.80	0.68	0.95	9.92E-03					
SMC1A	-0.26	0.77	0.64	0.92	4.51E-03					
TMC6	-0.17	0.84	0.72	0.99	3.92E-02					
PAXIP1	-0.19	0.83	0.70	0.98	3.22E-02					
MFAP2	0.24	1.27	1.07	1.50	6.88E-03					
MSI2	-0.27	0.77	0.65	0.91	2.64E-03	-0.30	0.74	0.61	0.90	2.24E-03
KYNU	0.20	1.22	1.03	1.46	2.35E-02	0.29	1.34	1.11	1.62	2.60E-03
POP1	-0.21	0.81	0.69	0.96	1.53E-02					
WDR4	-0.20	0.82	0.69	0.98	2.44E-02					
ZNF200	-0.18	0.84	0.70	1.00	4.63E-02					
SUSD1	0.17	1.19	1.00	1.41	4.68E-02					
SAC3D1	-0.19	0.83	0.70	0.98	2.64E-02					
MTPAP	-0.19	0.83	0.71	0.98	2.35E-02					
ZMYM1	-0.18	0.83	0.70	0.98	3.04E-02					
CXorf38	-0.19	0.83	0.70	0.99	3.75E-02					
PSMD12	-0.18	0.84	0.71	0.99	4.00E-02					
ACLY	0.17	1.19	1.03	1.38	2.06E-02	0.45	1.58	1.34	1.86	7.02E-08
NSD2	-0.19	0.82	0.69	0.98	3.10E-02					
SLC1A5	-0.18	0.84	0.71	0.98	2.75E-02					
KIF21B	-0.24	0.79	0.66	0.94	9.64E-03	-0.30	0.74	0.62	0.89	1.19E-03
ZNF121	-0.22	0.80	0.69	0.94	7.89E-03					
GPX8	0.24	1.27	1.08	1.49	4.29E-03					
REPIN1	-0.26	0.77	0.65	0.91	2.30E-03					
PRR5	-0.18	0.84	0.72	0.98	2.24E-02					
ZNF557	-0.27	0.76	0.64	0.90	1.67E-03					
VSNL1	-0.17	0.84	0.72	0.99	3.37E-02					
ZNF786	-0.23	0.80	0.68	0.94	6.90E-03					
PLA2G15	0.19	1.21	1.02	1.43	2.50E-02					
ZNF440	-0.19	0.83	0.70	0.98	2.76E-02					
SLC12A7	-0.23	0.80	0.68	0.94	8.33E-03	-0.24	0.79	0.65	0.96	1.64E-02
ZNF823	-0.27	0.76	0.64	0.90	1.40E-03	-0.22	0.80	0.66	0.98	2.88E-02
THOP1	-0.22	0.80	0.67	0.95	1.01E-02					
TNFAIP2	-0.20	0.82	0.70	0.97	1.83E-02					
PLAGL1	0.16	1.18	1.01	1.38	4.19E-02					
ZNF331	0.22	1.25	1.05	1.49	1.06E-02					

Risk score = (0.56321) × Expression Value (*KIF15*) + (−0.24228) × Expression Value (*FEN1*) + (−0.25944) × Expression Value (*ZFP69B*) + (−0.23267) × Expression Value (*SP6*) + (0.18278) × Expression Value (*SPARC*) + (−0.3753) × Expression Value (*TTF2*) + (−0.29728) × Expression Value (*MSI2*) + (0.29177) × Expression Value (*KYNU*) + (0.45439) × Expression Value (*ACLY*) + (−0.30168) × Expression Value (*KIF21B*) + (−0.23641) × Expression Value (*SLC12A7*) + (−0.22233) × Expression Value (*ZNF823*).

**Figure 4:**
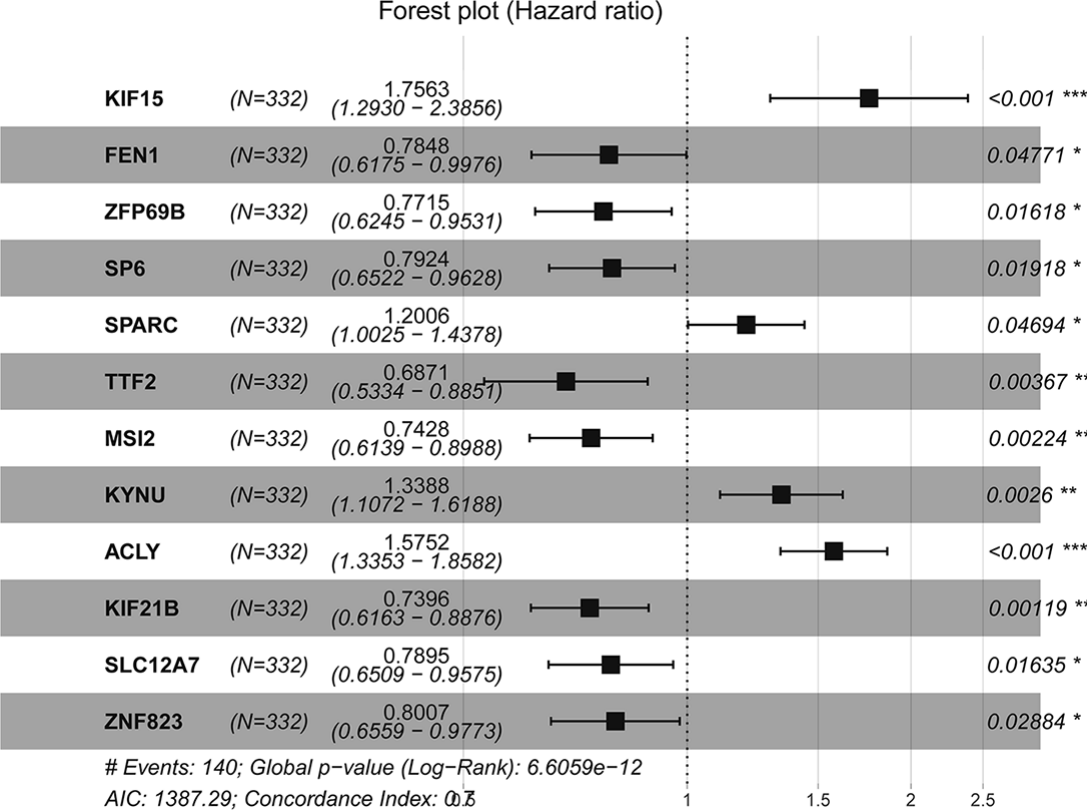
Forest plot of hazard ratios for overall survival of gastric cancer (C-index = 0.7).

**Figure 5:**
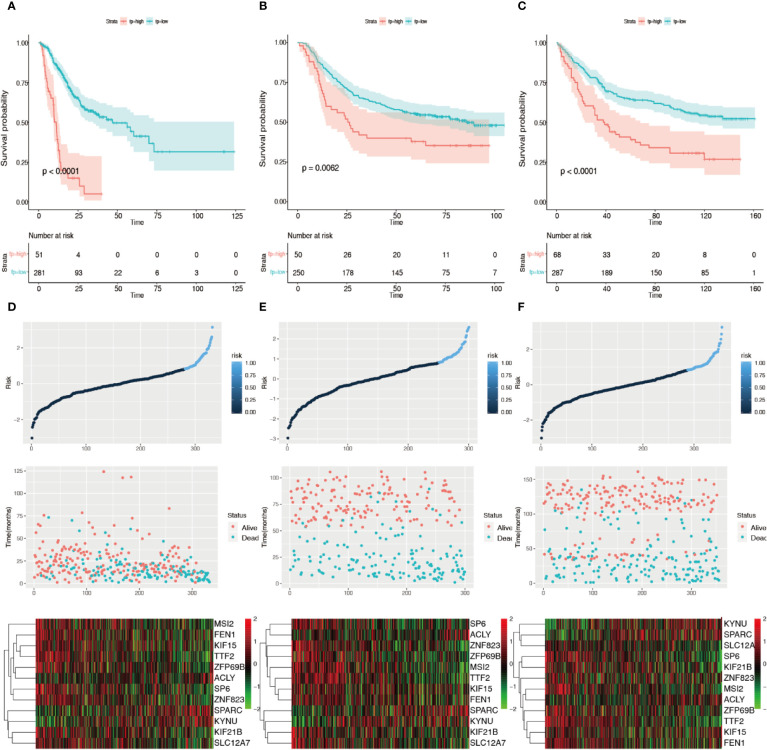
Validation of the 12 prognosis-related gene risk score system. **(A)** Kaplan–Meier survival curves of the training group using 0.82 as the cutoff value. **(B)** Kaplan–Meier survival curves of the GSE25544 dataset using 0.82 as the cutoff value. **(C)** Kaplan–Meier survival curves of the GSE83344 based on the 12 prognosis-related genes using 0.82 as the cutoff value. **(D)** 12-gene signature risk score panel for the gene signature in the training group. **(E)** 12-gene signature risk score panel for the gene signature in GSE25544. **(F)** 12-gene signature risk score panel for the gene signature in GSE83344.

The forest plot of hazard ratios (HRs) is shown in **Figure 4** using TCGA datasets. The HRs of *KIF15* (HR = 1.756), *SPARC* (HR = 1.201), *KYNU* (HR = 1.339), and *ACLY* (HR = 1.575) were greater than 1; however, the HRs of *FEN1* (HR=0.784)*, ZFP69B* (HR = 0.772)*, SP6* (HR = 0.792), *TTF2* (HR = 0.687)*, MSI2* (HR = 0.743), *KIF21B* (HR = 0.740)*, SLC12A7* (HR = 0.790), and *ZNF82* (HR = 0.801) were less than 1. We further discovered that the best cutoff risk score to differentiate low-risk from high-risk groups was 0.82. The risk score for each individual were calculated and was categorized into two groups according to the cutoff value. Kaplan-Meier analysis showed that there was a significant difference between high-risk (n=51) and low-risk patients (n=281) in the training dataset (log-rank test, p < 0.0001, **Figure 5A**). Risk stratification, survival information, and expression values of 12 genes of 332 patients were shown in the risk score panel. We observed that both the survival information and the 12-gene expression of patients in the high-risk group varied from those in the low-risk group (**Figure 5D**). Using GSE62254 and GSE84433 as validation datasets, the survival curves (**Figures 5B, C**) and the risk score panels (**Figures 5E, F**) between the high-risk groups and the low-risk groups were distinctively different. The results in the validation datasets were similar to those in the training dataset, therefore robustness of the 12-gene signature risk score system in predicting sample risk was supported.

### Cerna Network Construction

**Figure 6:**
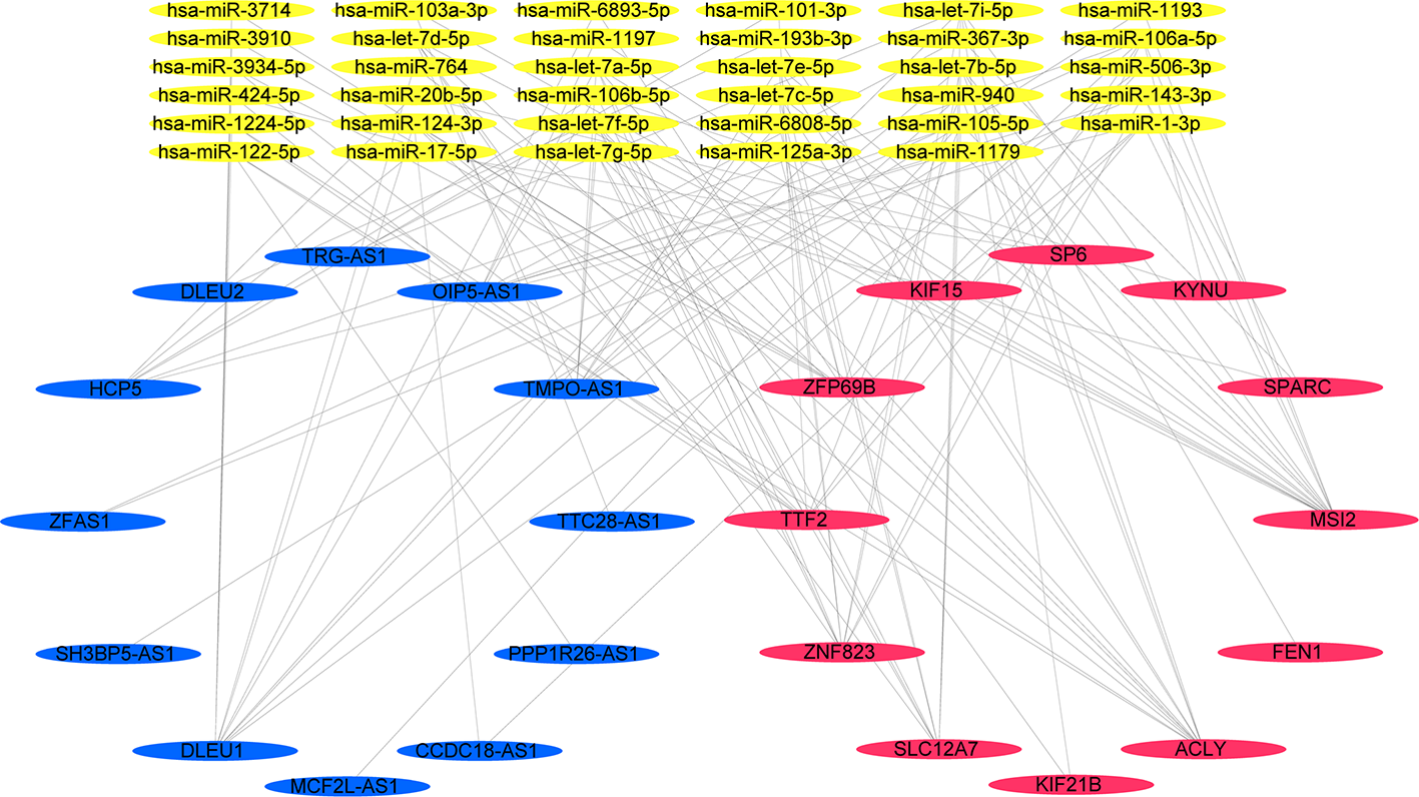
A lncRNA–miRNA–mRNA ceRNA network constructed from 12 prognosis-related genes.

We narrowed interesting lncRNAs to an intersection of lncRNAs predicted by the 12-mRNA model and differentially expressed lncRNAs, with eight upregulated lncRNAs (OIP5-AS1, MCF2L-AS1, TMPO-AS1, HCP5, DLEU1, PPP1R26-AS1, DLEU2, and ZFAS1) and four downregulated lncRNAs (SH3BP5-AS1, CCDC18-AS1, TTC28-AS1, and TRG-AS1) identified. In total, 35 miRNAs and 12 mRNAs in the risk score system, and 12 differently expressed lncRNAs were identified to create the ceRNA network. The correlation of 12 mRNAs and 12 lncRNAs was confirmed by both the Pearson correlation test and the hypergeometric test (**Table 3**). A ceRNA network was constructed using 86 dysregulated lncRNA–mRNA–ceRNA pairs (**Figure 6**).

**Table 3:** Pearson correlation tests and hypergeometric tests of the candidate lncRNA-miRNA-mRNA competing endogenous RNA pairs.

Genes	lncRNAs	miRNAs	hyperPValue	Correlation	corPValue
SPARC	OIP5-AS1	hsa-miR-367-3p, hsa-miR-424-5p, hsa-miR-143-3p	3.24E-04	1.23E-01	4.58E-03
SLC12A7	MCF2L-AS1	hsa-miR-105-5p	3.85E-02	2.67E-01	4.97E-09
SLC12A7	TMPO-AS1	hsa-let-7c-5p, hsa-let-7b-5p, hsa-let-7a-5p, hsa-let-7f-5p, hsa-let-7d-5p, hsa-let-7i-5p, hsa-let-7e-5p, hsa-let-7g-5p	3.17E-11	1.07E-01	1.21E-02
KYNU	HCP5	hsa-miR-106b-5p, hsa-miR-106a-5p	2.96E-04	2.33E-01	3.11E-07
TTF2	DLEU1	hsa-miR-106b-5p, hsa-miR-124-3p, hsa-miR-1224-5p, hsa-miR-3934-5p	2.61E-02	4.08E-01	1.31E-19
TTF2	HCP5	hsa-miR-106b-5p, hsa-miR-1-3p, hsa-miR-101-3p, hsa-miR-106a-5p	4.91E-04	1.35E-01	2.18E-03
TTF2	PPP1R26-AS1	hsa-miR-122-5p	3.38E-02	2.94E-01	1.24E-10
TTF2	CCDC18-AS1	hsa-miR-124-3p, hsa-miR-1-3p	3.32E-03	1.47E-01	9.55E-04
TTF2	MCF2L-AS1	hsa-miR-105-5p	3.38E-02	1.78E-01	7.73E-05
KIF21B	TMPO-AS1	hsa-let-7f-5p, hsa-let-7i-5p	1.44E-03	9.07E-02	2.78E-02
KIF21B	TRG-AS1	hsa-let-7f-5p, hsa-let-7i-5p	1.44E-03	2.88E-01	3.04E-10
ACLY	PPP1R26-AS1	hsa-miR-122-5p	1.75E-02	1.79E-01	6.98E-05
ACLY	DLEU1	hsa-miR-124-3p, hsa-miR-1224-5p, hsa-miR-940	2.05E-02	1.05E-01	1.32E-02
ACLY	TMPO-AS1	hsa-let-7c-5p, hsa-let-7b-5p, hsa-let-7a-5p, hsa-let-7f-5p, hsa-let-7d-5p, hsa-let-7i-5p, hsa-let-7e-5p, hsa-let-7g-5p	4.06E-14	1.47E-01	9.60E-04
MSI2	MCF2L-AS1	hsa-miR-105-5p	1.55E-02	1.77E-01	8.64E-05
MSI2	CCDC18-AS1	hsa-miR-1-3p	4.59E-02	1.32E-01	2.68E-03
MSI2	SH3BP5-AS1	hsa-miR-1193	1.55E-02	8.47E-02	3.70E-02
MSI2	OIP5-AS1	hsa-let-7a-5p, hsa-miR-105-5p, hsa-miR-1179, hsa-miR-1197, hsa-miR-143-3p	5.14E-07	9.54E-02	2.20E-02
MSI2	TTC28-AS1	hsa-miR-103a-3p, hsa-miR-106a-5p	1.39E-03	1.11E-01	9.48E-03
MSI2	TMPO-AS1	hsa-let-7c-5p, hsa-let-7b-5p, hsa-let-7a-5p, hsa-let-7f-5p, hsa-let-7d-5p, hsa-let-7i-5p, hsa-let-7e-5p, hsa-let-7g-5p, hsa-miR-1179	2.04E-17	1.26E-01	3.86E-03
KIF15	DLEU2	hsa-miR-193b-3p	7.75E-03	6.38E-01	1.08E-52
KIF15	HCP5	hsa-miR-1-3p	1.85E-02	2.13E-01	2.96E-06
FEN1	TMPO-AS1	hsa-let-7b-5p	3.45E-02	6.20E-01	4.29E-49
ZFP69B	DLEU1	hsa-miR-3714, hsa-miR-125a-3p, hsa-miR-124-3p, hsa-miR-506-3p, hsa-miR-764, hsa-miR-3934-5p, hsa-miR-6893-5p, hsa-miR-3910, hsa-miR-940, hsa-miR-6808-5p	3.02E-08	2.33E-01	3.19E-07
SP6	HCP5	hsa-miR-20b-5p, hsa-miR-17-5p	1.51E-03	1.02E-01	1.58E-02
ZNF823	TMPO-AS1	hsa-let-7c-5p, hsa-let-7b-5p, hsa-let-7a-5p, hsa-let-7f-5p, hsa-let-7d-5p, hsa-let-7i-5p, hsa-let-7e-5p, hsa-let-7g-5p	1.45E-14	3.04E-01	2.57E-11
ZNF823	ZFAS1	hsa-miR-106a-5p, hsa-miR-143-3p	7.92E-03	2.01E-01	9.84E-06

## Discussion

Currently, although there have been some investigations concentrating on non-coding RNA-involving gene expression regulation networks in gastric cancer, our research is the first to employ WGCNA to produce a co-expression network of lncRNAs–miRNAs–mRNAs in gastric cancer. Compared with the previous risk score-based system in gastric cancer (Cho et al., 2011; Cheong et al., 2018; Duan et al., 2019; Hu et al., 2019), the advantages of our ceRNA network are that it provided 12 both cancer-associated and prognosis-related genes and was constructed using rigorous calculation of the ceRNA regulation relationships.

*KIF15* is a gene involved in immune diseases and cancer progression. Whether it is mutated is related to the sensitivity of immune checkpoint inhibitors. Győrffy et al. reported that in both wild-type PIK3CA patient groups, individuals with *KIF15* mutations displayed substantially increased expression of PD-L1, whereas those without mutations displayed decreased expression (Menyhart et al., 2018). Zhang et al. found that knockdown of *KIF15* resulted in mitochondrial damage and ROS-JNK-p53 axis activation, thus promoting apoptosis and inhibiting cell proliferation in gastric cancer cells (Tao et al., 2020). The DNA replication and repair pathway is an important mechanism in gastric cancer. *FEN1* plays an important role in apoptotic fragmentation of DNA, maintenance of telomere stability, and rescue of stalled replication forks (Shen et al., 2005; Zheng et al., 2011). Unsurprisingly, genetic variants and changes in the expression of *FEN1* will alter patients’ sensitivity to chemotherapy and prognosis (Liu et al., 2012; Wang et al., 2014; Xie et al., 2016). The role of *SPARC* in gastric cancer has not been fully elucidated, although its diagnostic and prognostic value has been confirmed by multiple studies (Zhao et al., 2010; Liao et al., 2018). *SPARC* can act as both an inhibitor and promoter in cancer (Tai and Tang, 2008; Said, 2016; Li et al., 2019); the real effect of SPARC can be altered by other genes owing to its epistatic effects (Chen et al., 2018). However, some oncologists have shown that *SPARC* is mostly produced by gastric cancer-associated fibroblasts rather than gastric cancer cells (Ma et al., 2018; Ma et al., 2019). Thus, the concrete mechanism of *SPARC* in gastric cancer needs further investigation. *MSI2* is an oncogene associated with differentiation, resulting in the preservation of cancer stem cells. According to a previous study based on microarray and RT-PCR, *MSI2* expression increased slightly relative to normal tissue, but it is still used as a biomarker for gastric cancer (Emadi-Baygi et al., 2013; Yang et al., 2019). SLC12A7 (Solute Carrier Family 12 Member 7) acts as a potassium/chloride co-transporter for maintaining a stable osmotic pressure, which can be activated by insulin-like growth factor (IGF) resulting in cell invasion and progression in breast cancer, adrenocortical cancer, cervical cancer, and ovarian cancer (Shen et al., 2004; Hsu et al., 2007; Chen et al., 2009; Brown et al., 2019). It has been reported that amplification of *SLC12A7* is mainly within *HER2*− patient samples in gastric cancer, while the precise mechanism of *SLC12A7* is still unknown (Zhou et al., 2018). ACLY is the integral enzyme responsible for the formation of cytosolic acetyl-CoA, and high expression of *ACLY* was shown to be associated with a poor prognosis (Qian et al., 2015). Citrate and inhibitors of ACLY can reduce its expression, thus protecting against gastric cancer cell progression (Guo et al., 2016; Icard et al., 2020). In addition, *ACLY* can be downregulated by miR-133b *via* PPARγ (Cheng et al., 2017) and lncRNA FLJ22763 (Zhang et al., 2019) in gastric cancer.

The functions of *ZNF823*, *ZFP69B*, *SP6*, *KYNU*, *KIF21B*, and *TTF2* have not been reported in gastric cancer. KYNU is a pyridoxal-5´-phosphate dependent enzyme that catalyzes the cleavage of kynurenine into anthranilic acid (AA) and the cleavage of 3-hydroxykynurenine (3-HK) into 3-hydroxyanthranilic acids (3-HAA) (Badawy, 2017). Drugs have been developed to regulate the expression of KYNU to suppress tumor growth through the Kynurenine pathways, such as in breast cancer (Liu et al., 2019; Liu et al., 2020) and melanoma (Rad et al., 2019). It has been demonstrated that TTF2 is able to terminate RNA polymerase II transcription, which has an important function in promoting chromosome segregation and altering protein-DNA interactions (Jiang and Price, 2004; Jiang et al., 2004; Cheng et al., 2012). KIF21B is an ATP-dependent microtubule-based motor protein that participates in the intracellular transfer of membranous organelles. *KIF21B* is a potential oncogene that resists the induction of apoptosis and facilitates malignant tumorigenesis, tumor development, intrusion, and metastasis. Patients with high expression of *KIF21B* have been demonstrated to have a poorer prognosis in hepatocellular carcinoma (Zhao et al., 2020) and non-small cell lung cancer (Sun et al., 2020). *SP6* is an important gene that regulates odontogenesis, belonging to a family of transcription factors that contain 3 classical zinc finger DNA-binding domains(Aurrekoetxea et al., 2016; Smith et al., 2020; Nakamura et al., 2020).

In summary, we identified a survival-related gene-based ceRNA network using the WGCNA algorithm, and the constructed lncRNA–miRNA–mRNA ceRNA interactive network will probably provide a basis for additional inspection of the regulatory mechanisms of gastric cancer. Gastric cancer has high heterogeneity among its different histological and molecular subtypes. Thus, while we have selected the expression profiles with the largest sample size that we could obtain currently, we must admit that further experimental works and large cohorts are needed to verify our results and elucidate the prognostic value of ceRNA networks in gastric cancer.

## Data Availability Statement

The datasets analyzed for this study can be found in The Cancer Genome Atlas (TCGA) data portal (https://portal.gdc.cancer.gov/), the Genotype-Tissue Expression (GTEx) portal. (https://www.gtexportal.org/home/index.html), and the Gene Expression Omnibus (GEO) repository (https://www.ncbi.nlm.nih.gov/geo/).

## Author Contributions

XZ and YX contributed to study conception. XZ, XW, LZ, and HZ contributed to data collection and analysis. XZ, WL, BW, LX, and YT contributed to manuscript writing. All authors contributed to the article and approved the submitted version.

## Funding

The study was supported by the CAMS Initiative for Innovative Medicine (2016-I2M-1-007) and China International Medical Foundation (CIMF-F-H001-314).

## Conflict of Interest

The authors declare that the research was conducted in the absence of any commercial or financial relationships that could be construed as a potential conflict of interest.
